# Supplementary material for: Sagittal spinal morphotype assessment in 8 to 15 years old Inline Hockey players
Source: PeerJ. 2020 Jan 2;8:e8229. doi: 10.7717/peerj.8229 (PMC6942677; doi:10.7717/peerj.8229)
Supplement: Supplemental Information 2 [file peerj-08-8229-s002.docx]

Codebook raw data

1. N = participant number
2. Deporte = sport
3. Sexo = gender (1 boy; 2 girl)
4. Edad = age
5. Peso = weight
6. Talla = height
7. IMC = BMI
8. Categoria = Category (1 U6, 2 U11, 3 U13, 4 U16)
9. NivelComp = Competition level
10. Puesto = player position (1 goalkeepers, 2 defence, 3 striker, 4 defence-striker)
11. EESS Dom = dominant arm (1 right, 2 left)
12. EEII Dom = dominant leg (1 right, 2 left)
13. BIP dorsal = dorsal angle in standing position
14. Clasif BIP dorsal = dorsal classification in standing position (1 reduced kyphosis, 2 normal kyphosis, 3 hyperkyphosis)
15. BIP lumbar = lumbar angle in standing position
16. Clasi BIP lumbar = lumbar classification in standing position (1 reduced lordosis, 2 normal lordosis, 3 hyperlordosis)
17. SED dorsal = dorsal angel in slump sitting
18. Clasif SED dorsal = dorsal classification in slump sitting (1 reduced kyphosis, 2 normal kyphosis, 3 hyperkyphosis)
19. SED lumbar = lumbar angle in slump sitting
20. Clasif SED lumbar = lumbar classification in slump sitting (1 reduced kyphosis, 2 normal kyphosis, 3 hyperkyphosis)
21. Morfotiposagitalintegral dorsal and V. Codificacion D = sagittal thoracic morphotype and codification (1 hypokyphotic attitude in standing position, 2 hypomobile kyphosis, 3 normal kyphosis, 4 hyperkyphosis in standing position, 5 functional hyperkyphosis in static, 6 functional hyperkyphosis in dynamic, 7 total functional hyperkyphosis, 8 hyperkyphosis in static, 9 hyperkyphosis in dynamic, 10 total hyperkyphosis)
22. Morfotiposagitalintegral Lumbar and X. Codificacion L = sagital lumbar morphotype and codification (1 structured lumbar kyphosis, 2 lumbar kyphosis, 3 hypolordotic attitude, 4 normal lordosis, 5 functional hyperkyphosis in static, 6 functional hyperkyphosis in dynamic, 7 total functional hyperkyphosis, 8 hyperlordotic attitude, 9 functional lordosis, 10 lumbar hypermobility)
23. DDP L-Hfx = Lumbo-horizontal angle
24. Clasif DDP L-Hfx = Lumbo-horizontal angle classification (<100 normal, 110-118 slight posterior pelvic tilt, >119 moderate posterior pelvic tilt)
